# Supplementary material for: Identification of photoperiod-induced specific miRNAs in the adrenal glands of Sunite sheep (Ovis aries)
Source: Front Vet Sci. 2022 Jul 22;9:888207. doi: 10.3389/fvets.2022.888207 (PMC9354845; doi:10.3389/fvets.2022.888207)
Supplement: Supplementary file 1 [file Data_Sheet_1.ZIP › Supplementary file/miRDeep2 file.zip!/miRDeep2 file/Know_Structure/SP42A3/oar-mir-181a-2.pdf]

miRBase precursor : oar-mir-181a-2
Total read count : 14348
oar-miR-181a read count : 14186
remaining reads : 162

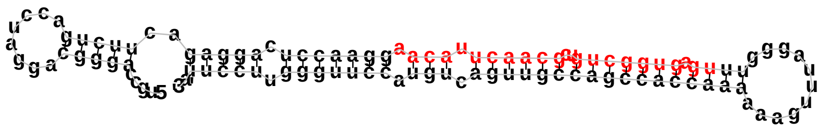

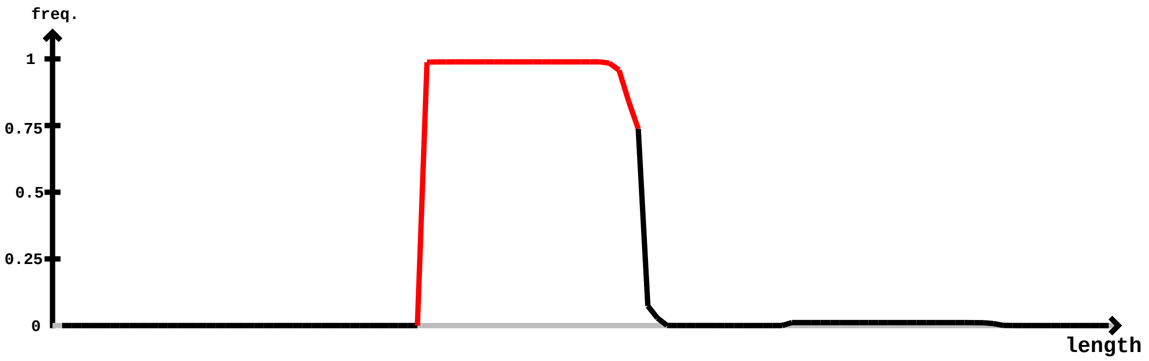


```
                                     oar-miR-181a
5'- ugccagggcaggauccagucuucagaggacuccaaggaacauucaacgcugucggugaguuugggauuugaaaaaaccaccgaccguugacuguaccuuggguuccuua   -3'   exp
    ....(((((........)))))..(((((.(((((((.(((.((((((..(((((((.((((...........))))))))))))))))).))).))))))).))))).    reads   mm   sample
    .....................................aacauucaacgcugucggu.....................................................    69      0    seq
    .....................................aacauucaacgcugucggug....................................................    377     0    seq
    .....................................aacauucaacgcugucgguga...................................................    1673    0    seq
    .....................................aacauucaacgcugucggugag..................................................    1484    0    seq
    .....................................aacauucaacgcugucggugagu.................................................    9512    0    seq
    .....................................aacauucaacgcugucggugaguu................................................    651     0    seq
    .....................................aacauucaacgcugucggugaguuu...............................................    398     0    seq
    .....................................aacauucaacgcugucggugaguuug..............................................    3       0    seq
    .....................................aacauucaacgcugucggugaguuugggauu.........................................    1       0    seq
    ......................................acauucaacgcugucgguga...................................................    2       0    seq
    ......................................acauucaacgcugucggugag..................................................    1       0    seq
    ......................................acauucaacgcugucggugagu.................................................    12      0    seq
    ......................................acauucaacgcugucggugaguuu...............................................    1       0    seq
    .......................................cauucaacgcugucggugagu.................................................    1       0    seq
    ........................................auucaacgcugucggugagu.................................................    2       0    seq
    ..........................................................................aaccaccgaccguugacugu...............    1       0    seq
    ..........................................................................aaccaccgaccguugacugua..............    1       0    seq
    ..........................................................................aaccaccgaccguugacuguac.............    1       0    seq
    ..........................................................................aaccaccgaccguugacuguacc............    1       0    seq
    ...........................................................................accaccgaccguugacugu...............    4       0    seq
    ...........................................................................accaccgaccguugacugua..............    5       0    seq
    ...........................................................................accaccgaccguugacuguac.............    42      0    seq
    ...........................................................................accaccgaccguugacuguacc............    96      0    seq
    ...........................................................................accaccgaccguugacuguaccu...........    9       0    seq
    ...........................................................................accaccgaccguugacuguaccuu..........    1       0    seq
```
